# Pelvic Lesions in Relation to Their Distinctive Effects upon Mental Disturbances1Read before the Buffalo Academy of Medicine, November 26, 1901.

**Published:** 1902-02

**Authors:** A. T. Hobbs

**Affiliations:** Late of London, Ont.; Now Superintendent Homewood Retreat, Guelph, Ont.


### BUFFALO MEDICAL JOURNAL.

VOL. XLI.—LVII. FEBRUARY, 1902. NO. 7.

### ORIGINAL COMMUNICATIONS.

### Pelvic Lesions in Relation to Their Distinctive Effects upon Mental Disturbances.[1]

BY A. T. HOBBS, M. D., late of London, Ont.
Now Superintendent Homewood Retreat, Guelph, Ont.

THE gradual evolution of the treatment of the insane unfolds factors in etiology that hitherto were unsuspected or given but little credence to as possible disturbers, either directly or indirectly, of the mental equilibrium of the sane. The surgical treatment of organic disease in patients mentally deranged was followed by mental phenomena so marked that the inference was reached that there must exist a relationship between certain physical lesions and mental disease, as the removal of the former was succeeded by an improvement or subsidence of the latter. This occurred so frequently that it could not be dismissed as mere coincidence,but it positively determined that these bodily lesions were in themselves responsible for the initiation or maintenance of insanity per se.

Until within recent years female lunatics received precisely the same attention and treatment as that accorded to male lunatics. That insane females possessed either an ovary or an uterus was either overlooked or ignored, and the possibility of either of these sequestered organs being grossly diseased seems never to have been contemplated by those into whose charge was committed the care of the insane. In explanation it must be said that without close observation or systematic gynecological investigation it was practically impossible to definitely state that pelvic disease existed, much less could be diagnosticated in a female lunatic. For this reason and because of the woman's mental infirmity with its accompanying delusion, or mania, or stupor or

1. Read before the Buffalo Academy of Medicine, November 26, 1901.

dementia which rendered her unable to rationally complain of physical distress as would a sane woman, it was hard to convince the majority of alienists that 15, or 20, or 25 per cent. of all insane women were suffering from one or more pathological lesions of the organs of the reproductive system.

A consideration of the statistics of institutions devoted to the care of the insane will show, by comparison of sexes and of their civil state that among their lunatic populations there are more single than married men and more married than single women. Why is there this preponderance of the single in the one sex and of the married in the other? The solution of this, I believe, is that single men lead more irregular lives than married men and as a result are more liable to dissipations and exposures leading up to the disease with subsequent mental as well as physical collapse, also that married women preponderate over the single of their sex, owing to their liability to injury and disease entailed by maternity with its sequence of ill health and nerve depreciation ending up so frequently in mental degeneration. That this is true, as experience has shown, should it not emphasise the necessity of a systematic examination of at least of all married female insane when under treatment to determine the presence or absence of gynecic complication, whether the patients are resident or not of institutions or sanitariums?

The result of pelvic examination of a large proportion of the female population at the asylum for insane, London, Ont., disclosed at least the presence of organic disease or abnormalities in 25 per cent.

The gynecological examinations were uniformally conducted with the aid of an anesthetic. Experience has taught us that the most suitable and reliable anesthetic for the insane is ether, preceded by the inhalation of nitrous oxide gas. This is the only method of any value of arriving at a proper diagnosis of the existence and nature of disease of the pelvic organs among the female insane.

As a result of these investigations it was found that 253 out of 1,000 females, (who were residents of the institution during the past six years,) had some pelvic disease or abnormality that needed gynecological treatment. Medical and other treatment only temporised with these lesions and was found difficult to carry out and non-productive of result. The only success in combating these diseases was that obtained through surgical means.

The surgical methods employed were those that are in daily use by all reputable surgeons for treatment of similar lesions in the sane. Many a patient required two or more operations to complete the treatment in her case.

To ascertain the proper value of the results succeeding the removal of the different lesions the cases will be classified into groups, the principal gynecological lesion in each patient determining the position in the classification.

*Ovarian Diseases.*—The total number of cases who received treatment for disease of the ovaries and tubes was 41. The treatment in each necessarily varied according to the disease or the complication present. To accomplish this it was found necessary to perform in seven, hysterectomies,—four by the abdominal route and three per vaginam; in twenty-five cases, single or double oöphorectomy was done and in the remaining nine a part of one or both ovaries was preserved, after removal of the diseased portions. Following these operations for ovarian disease there occurred two deaths or 5 per cent., both dying of complicating pneumonia, one on the seventh and the other on the twelfth day after operation. Good physical recoveries resulted in the remaining 39, or 95 per cent.

The subsequent mental history in the 39 patients who survived the operation was very good. The time of mental recovery varied from three months to one year.

The mental classification is summarised as follows:

| | Cases. | Recoveries. |
|---|---|---|
| Acute mania | 11 | 7 |
| Chronic mania | 23 | 9 |
| Epileptic mania | 2 | 0 |
| Folie circularie | 2 | 1 |
| Psychocoma | 1 | 1 |
| Acute melancholia | 3 | 2 |

This gives a total of 20 recoveries or 49 per cent., The duration of the insanity in those 20 averaged eighteen months. Over and above this there were 10 patients, or 25 per cent., who showed a distinct mental improvement, although the average length of insanity in these ten exceeded three years.

The history of these ovarian cases disclosed heredity in 16 or 39 per cent. From this it will be seen that the introduction of modern surgery immensely benefited 30 women, representing 73 per cent. of the ovarian cases, by the reduction of disease tissue or of the removal of the entire organ whenever found necessary.

*Abnormally Displaced Uteri.*—It was found on examination of 66 patients that the main lesion presented was a displaced uterus. The abnormal position of this organ varied from simple retroversion to complete procidentia. To correct the pathological positions of this organ it was found necessary to shorten the round ligaments in 54 patients, to suspend the uterus per ventrum in 7, as well as to perform total extirpation in 7 others where the procidentia was complete.

These patients did not all do well, as death succeeded operation in two; one dying from secondary hemorrhage induced by the patient pulling out the ligatures, and the other from bedsores two months after treatment. Both of these occurred after vaginal hysterectomy.

A synopsis of the mental condition and recovery-rate of these 66 patients is tabulated below.

| | Cases. | Recoveries. |
|---|---|---|
| Acute mania | 20 | 15 |
| Chronic mania | 22 | 3 |
| Epileptic mania | 1 | 0 |
| Puerperal mania | 7 | 4 |
| Acute melancholia | 9 | 5 |
| Chronic melancholia | 1 | 1 |

From this table it will be seen that the mental condition was restored in 28 or 42 per cent., with an average duration of insanity of 1 year and 10 months. Besides these recoveries in 15 others or 23 per cent., the mental condition was more or less improved after correcting the displaced organ. This makes a total of 43 patients or 65 per cent. whose condition, both physically and mentally, responded to proper treatment. Of these 66 cases who had a mal-position of the uterus, it was found that 32 or 48 per cent. were tainted by hereditary insanity.

*Tumors—Malignant and Benign.*—Gynecological examination of 16 insane women disclosed as a complication of their insanity an acquired growth. Of these 9 had fibroid tumors of the uterus, 2 showed cervical epitheliomas, 1 a sarcoma of the body of the uterus, 2 had tubercular disease of the pelvic organs, and 2 had inflammatory deposits in and around the uterus. For the treatment of these foreign bodies there where performed 8 abdominal hysterectomies, 4 vaginal hysterectomies, 1 myomectomy, and 3 celiotomies, with the use of saline lavage in the tubercular cases.

Following operation in these 16 patients there resulted 1

death from exhaustion on the third day. The other 15, however, made good physical recoveries.

As to the mental features and number of recoveries the accompanying table will show:

| | Cases. | Recoveries. |
|---|---|---|
| Acute mania | 1 | 1 |
| Chronic mania | 11 | 1 |
| Epileptic mania | 1 | 0 |
| Chronic melancholia | 3 | 0 |

It will be seen by this analysis that only 2, or 12 per cent., recovered their reason subsequent to the removal of these physical lesions. The average duration of insanity in these 2 recoveries prior to operation was three years. There were 6 others, representing 37 per cent., whose mental status was improved. These latter, however, showed an average duration of insanity of over five years. Only 3 out of the 16 or 19 per cent. disclosed any heredity.

*Diseases or Injuries of Uterine Cervices.*—In 60 patients the main lesion which demanded surgical relief was a diseased or injured cervix. Nearly all of these cases were complicated by either a sub-involuted uterus or an endometritis. In 19 of these 60 cases there was, in addition to the cervical lesion, a complete or incomplete tear of the perineum. For the necessary relief of the diseased cervices there was carried out 52 amputations, 5 trachelorrhaphies and 3 underwent treatment by the method described by Dudley for the relief of stenosis of the internal os. Restoration to bodily health occurred in all. The accompanying table shows the mental state and the recovery rate of these 60 patients:

| | Cases. | Recoveries. |
|---|---|---|
| Acute mania | 17 | 12 |
| Chronic mania | 30 | 5 |
| Puerperal mania | 3 | 0 |
| Epileptic mania | 1 | 0 |
| Folie circularie | 2 | 0 |
| Chronic melancholia | 3 | 1 |
| Acute melancholia | 4 | 0 |

Following uterine and cervical treatment there was complete mental relief in 19 or 31 per cent. These showed an average insanity duration of 15 months. Besides these recoveries, 14 others or 23 per cent. improved mentally. A history of heredity in these cervical cases complicated 21 or 35 per cent. of the whole number.

*Diseases of the Uterine Body or its Lining Membrane.*—On examination of 52 patients it was deemed necessary to curette for the reduction of a sub-involuted uterus or the correction of an endometritis. Some of these when under previous observation were noted as being menorrhagic, or were suffering from dysmenorrhea. All these patients so treated improved in physical health. The mental results were as follows:

| | Cases. | Recoveries. |
|---|---|---|
| Acute mania | 23 | 14 |
| Chronic mania | 15 | 1 |
| Puerperal mania | 3 | 2 |
| Acute melancholia | 5 | 3 |
| Chronic melancholia | 4 | 3 |
| Puerperal melancholia | 2 | 2 |

From this table it will be seen that the mental recovery rate was 28 or 48 per cent. and their average length of insanity was 10 months. Besides this, 11 or 21 per cent. showed mental improvement, their insanity averaging three and a half years.

The question of hereditary showed itself in the histories of 15 or 29 per cent. of the 52 patients so treated.

*Injuries to the Perineal Body.*—Lacerations of the perineum of all degrees accompanied by varying prolapse of the vaginal walls was found to be the main lesion in 18 patients. Most of these cases had also to some extent subinvolution of the uterus, which was corrected at the same time as the repair of the trauma to the perineum. The surgical treatment benefited these patients materially, as was observed by the rapid improvement in general health and subsequent mental tone. The classification of the mental disease and subsequent history of these 18 cases was as follows:

| | Cases. | Recoveries. |
|---|---|---|
| Acute mania | 6 | 2 |
| Chronic mania | 4 | 0 |
| Puerperal mania | 2 | 1 |
| Acute melancholia | 4 | 3 |
| Chronic melancholia | 2 | 1 |

This summary shows that 7 or 39 per cent. recovered mentally succeeding the restoration of the injured perineum and the removal of its complications. The average duration of their insanity was only 9 months. Of the others 3 or 17 per cent. improved whose duration of mental enfeeblement exceeded 9 years. Heredity complicated only 4 or 22 per cent. of these 18 perineal cases.

It is necessary to state that the six divisions as given are somewhat imperfect, as often an ovarian case was complicated by a displaced uterus, or a displaced uterus had in addition a lacerated or diseased cervix, or a diseased cervix was often accompanied by a tear of the perineum. A more limited classification may be devised by taking the ovarian lesions as one, the uterine displacements and diseases of the body and cervix together as a second, the injuries of the perineum as a third, and the tumors as a fourth class. This arrangement will summarise as follows: Of ovarian disease there were 41 cases with 20 or 49 per cent. recoveries; of uterine lesions there were 178 cases with 72 or 40 per cent. of recoveries; of injuries to the vaginal outlet there were 18 cases, with 7 or 39 per cent. recoveries, and there were new growths in 16 cases with 2 or 12 per cent. recoveries. From this division it will be noted that the pelvic lesions having the greatest effect upon mental alienation were those in which there existed changes in the ovarian structure, causing an interference with the ovarian function. The next most potent pelvic factor was disease of uterus, and third most important were injuries to the via vaginalis, while fourth, and last, new growths did not seem to disturb mental stability, except in a small percentage of cases.

Two simple divisions may be made of the whole number by grouping together all ovarian and uterine lesions as inflammatory. This will show that out of 219 cases supposedly inflammatory that 92 or 42 per cent. returned to their normal mental state. Then group all the remainder, including new growths and injuries to the perineum as non-inflammatory and these will make a total of 34 cases, with a recovery rate of only 9 or 26 per cent.

An epitome of the various mental diseases, which were the main lesions in the 253 patients, illustrates briefly the phases of lunacy that were the most susceptible to alleviation on the removal of gynecic sources of irritation.

The acute insanities were naturally the most amenable mentally to favorable treatment of pelvic ailments, as the post-operative results already given have shown. In the acute mental affections the recoveries from mania took the lead with a percentage of 61, then followed melancholia with 58 per cent. of recoveries and puerperal insanity the last, with 53 per cent.

In the chronic cases melancholia yielded much better results to surgical treatment than mania, there being 46 per cent.

recoveries in the former to 25 per cent. recoveries of the latter. Of the 4 cases of folie circularie or circular insanity only 1 or 25 per cent. was mentally restored. Finally, of the 5 patients treated by these surgical methods 91 were complicated by a hereditary tendency or a percentage of 36.

In the former presentations of this work before medical societies some doubt was expressed as to the correctness of previous similar statistics, and we were said to be ultra-enthusiasts in this gynecological work. It was claimed that "we looked for disease and found it." In addition to this, there were some who endeavored through their criticisms to imply that we were guilty of unnecessary surgical interference.

These criticisms go beyond the Rubicon of legitimate argument and tend to cast odium upon the work that was done.

Regarding the want of faith in our statistics, I desire to place on record the following facts, which will confirm in a great measure the figures and deductions already given in detail.

For the past thirty years annual reports were presented to the Provincial government of all official statistics in connection with the varying movements of the population of London asylums. These statistics are substantially correct and are subject to government periodical supervision. The official records show that for the biquintennial period previous to the introduction of systematic surgical treatment the average annual rate of discharges of patients recovered and improved, calculated upon the admissions, was for the male residents 35.23 per cent. and for the female, 37.5 per cent.

For the third quintennial period, during which gynecological surgery was in vogue in addition to the ordinary methods of treatment, it was found that the annual rate of discharges among the men differed very little from that of the previous two quintennial periods, being 35, or 92 per cent. It was discovered, however, that the women during the third quintennial period had advanced from 37.5 per cent., the average of the previous ten years to 52.7 per cent., or a gain in the discharge rate among the women of 35 per cent. This was certainly due to the surgical treatment of pelvic disease which existed so largely among the female population, as the other methods of combating disease were practically the same as in previous years. An official analysis was also made concerning the number of readmissions of those who had been discharged during this third quintennial period. It was found that although many more women had been discharged than men the number of readmissions were the same for each sex, being 19 women and 19 men. This undoubtedly verifies the stability of the mental cases who recovered after the removal of complicating utero-ovarian disease and still further qualifies the assertion that these diseases play an important part in the etiology of insane women.

The charge that unnecessary surgical interference had been done in these cases is absurd as well as untrue, as prior to operation the patient's family physician was consulted and asked to be present at that operation. This invitation was often accepted and unqualified approval of the work done was uniformly expressed by these visiting physicians. In addition to this the written consent of the nearest relative was always obtained to even the most minor of operations. These were some of the safeguards which surrounded these patients from unnecessary surgical interference.

As to the charge that "we looked for disease and found it" is certainly a more favorable criticism than the ultra-conservative policy of some of the critics, and their adherence to antiquated methods of treatment still pursued by many of our alienists.

The value of gynecological as compared with general surgery is proved by the results obtained after operations for the radical cure of hernia. In 39 patients of both sexes who were afflicted with either a ventral, umbilical, inguinal, or femoral hernia, a radical cure was attempted, and I am pleased to say with almost uniform success as regards the obliteration of this physical lesion. The mental results succeeding the operation for hernias were almost *nil*, as no mental recovery occurred although decided improvement in the general tone of these patients was observed.

In conclusion, let me say that there should be no doubt in the minds of physicians—general and special—as to the benefits that would accrue from the introduction and proper observance of aseptic gynecological surgery in institutions devoted to the care of the insane. Also that the state should see that its wards are properly safeguarded against unnecessary operations, such as the removal of normal ovaries for their possible effect upon a distured mental condition. That this has been done occasionally by surgeons I have reason to know and the results have been decidedly harmful, not only to the patients but to the establishment of gynecology as one of the regular methods that should be employed in institutions where so many women are incarcerated and who without the aid that gynecology can give, are doomed to suffer untold misery as long as their existence endures.

